# A Glow before Darkness: Toxicity of Glitter Particles to Marine Invertebrates

**DOI:** 10.3390/toxics11070617

**Published:** 2023-07-16

**Authors:** Denis Moledo de Souza Abessa, Letícia França Albanit, Pedro Henrique Paixão de Moura, Vitória Soares Nogueira, Felipe Teixeira Santana, Kainã Fagundes, Maysa Ueda, Otto Patrão de Oliveira Muller, Caio Cesar-Ribeiro

**Affiliations:** 1 São Paulo State University—UNESP, Coastal Campus, Department of Biological and Environmental Sciences, São Vicente 11380-900, SP, Brazil; lealbanit@gmail.com (L.F.A.); pedro.paixao@unesp.br (P.H.P.d.M.); vitoria.nogueira@unesp.br (V.S.N.); ft.santana@unesp.br (F.T.S.); kaina.fagundes@unesp.br (K.F.); maysa.ueda@hotmail.com (M.U.); caiocribeiro@hotmail.com (C.C.-R.); 2 Central de Equipamentos Multidisciplinar (CEM), Universidade Federal do ABC UFABC; São Bernardo do Campo 09850-910, SP, Brazil

**Keywords:** microplastics, ecotoxicology, marine pollution, contaminants of emerging concern, chronic toxicity

## Abstract

Glitter particles are considered a model of microplastics, which are used in a wide range of products. In this study, we evaluated the toxicity of two types of glitter (green and white, with distinct chemical compositions) dispersions on the embryonic development of the sea urchins *Echinometra lucunte*, *Arbacia lixula*, and the mussel *Perna perna*. The Toxicity Identification and Evaluation (TIE) approach was used to identify possible chemicals related to toxicity. Glitter dispersions were prepared using 0.05% ethanol. The tested dispersions ranged from 50 to 500 mg/L. The white glitter was composed of a vinyl chloride–methyl acrylate copolymer. The effective concentrations of green glitter to 50% embryos (EC50) were 246.1 (235.8–256.4) mg/L to *A. lixula*, 23.0 (20.2–25.8) mg/L to *P. perna* and 105.9 (61.2–150.2) mg/L, whereas the EC50 of white glitter to *E. lucunter* was 272.2 (261.5–282.9) mg/L. The EC50 for *P. perna* could not be calculated; however, the lowest effect concentration was 10 mg/L—that was the lowest concentration tested. The filtered suspension of green glitter had Ag levels exceeding the legal standards for marine waters. TIE showed that metals, volatiles, and oxidant compounds contribute to toxicity. The results showed that glitter may adversely affect marine organisms; however, further studies are necessary to determine its environmental risks.

## 1. Introduction

Plastics represent the dominant fraction of marine litter, and less than 10% of the plastics used worldwide are recycled [1,2]. Approximately 367 million tons of plastics were produced in 2021, and this number is estimated to increase by 33 million tons by 2050 [3]. Therefore, plastic pollution has become an increasingly global concern [4,5], particularly of microplastics (MPs)—that is, plastic particles ranging between 1 μm and 5 mm [6], which constitutes a predominant form of plastic litter in the marine environment [7,8]. Plastics discharged into freshwater streams are often transported to the sea [9,10,11,12,13], where they accumulate in large quantities in both the water column and the sediments [14,15,16,17].

Microplastics of primary origin are those produced intentionally at the microscopic scale as precursors of other products or for direct use as abrasives in cleaning and aesthetic products [18]. They include microbeads, which are small plastic spheres that often range between 5 μm and 1 mm. They are composed of various plastic polymers [19] and are included in several personal care products [19,20,21]. Because microbeads are designed to be washed and carried into drains and their large-scale production, microbeads are considered potential contaminants in the marine environment [19].

However, according to Piccardo et al. [22] and Albanit et al. [18], there is another category of primary MPs that is almost completely ignored today: glitter particles. The glitter particle is formed by a set of plastic layers covered by thin metallic layers, similar to a sandwich, as described by [23]. These include a variety of small, plain, and reflexive particles used in craftwork, textiles, and cosmetic products [8]. These particles have some similarities to microbeads; however, they have not received appropriate attention from the scientific community and society as potential contaminants [8,18,22,23]. According to Provenza et al. [24], glitter may be a model for MPs and can be considered a “symbol of microplastic”, comprising of all the hazardous characteristics of MPs.

Glitter is widely used in makeup, clothes and fancies, carnival floats, body paints, nail polish, craft products, and other materials [18,25]. It was also included in a list of polymer-based materials that were intentionally produced as MPs, according to the European Chemicals Agency [26]. Glitter particles are carried into domestic wastewater and then released into natural aquatic environments [23]. Albanit et al. [18] also stated that urban drainage may represent an additional route for glitter entering the environment. Tagg and Ivar do Sul [23] stated that glitter could be a good indicator of sewage in marine waters. Raju et al. [27] estimated the daily input of glitter particles from a wastewater treatment plant to be 2.7–3.0 × 10^7^ particles/day.

Glitter particles have been reported in sewage sludge samples from riverbed sediments in North England [28,29], samples from a wastewater plant located in Norway [30], and sediments from UK rivers [28]. Moreover, as increasing amounts of MPs have been found in aquatic environments worldwide [31], the quantity of glitter may follow this trend. In a review, Yurtsever [8] identified studies reporting the presence of glitter in aquatic environments and addressed the potential environmental hazards associated with glitters.

However, glitter particles have historically been overlooked as environmental contaminants [2,22,23,24]. Although some recent studies have investigated the potential environmental impacts of glitter, very little information is available on the potential effects of glitter on marine biota, particularly neotropical organisms. Green et al. [32] compared the ecological impacts of conventional polyethylene terephthalate (PET) glitter (non-biodegradable) with glitters made of alternative materials on the biodiversity and functioning of freshwater ecosystems, and found that all types of glitters tested could cause adverse effects on aquatic ecosystems. More recently, Provenza et al. [24] reported that the ingestion of glitter by the bivalve *Mytilus galloprovincialis* induced biochemical alterations, whereas Piccardo et al. [22] reported that glitter leachates caused toxicity in several aquatic organisms. Pramanik et al. [2] observed that exposure to glitter particles caused several disturbances in adults of brine shrimp *Artemia salina*, while Albanit et al. [18], in a preliminary study with neotropical organisms, showed that glitter leachates could cause reduction of embryonic development in sand-dollars.

The toxicity associated with microplastics, including glitter, has been attributed the addition of a range of chemicals to plastic polymers during their production [18,25], although little is known about the composition and effects of plastic additives on aquatic biota [22]. According to these authors, such additives include stabilizers, flame retardants, antistatics, plasticizers, lubricants, and biocides. In the case of glitter particles, the authors also mentioned the relevance of dyes; however, the possible substances included in this category are not known. Therefore, efforts to determine the chemicals responsible for microplastics toxicity are relevant. The toxicity identification and evaluation (TIE) approach has been applied to complex matrices, such as effluents and sediments, to identify substances that contribute to toxicity [33,34]. The TIE approach involves a series of physical and chemical manipulations of samples that may cause a decrease, an increase, or transformations in the bioavailability of different toxic groups [35]. Such manipulations involve a set of treatments applied to a determined sample, such as aeration, filtration, the addition of chelants (EDTA) or reducing substances (sodium thiosulfate), C18 column, pH alteration, and the removal of ammonia [33,34,35,36,37]. They are followed by toxicity tests to detect the chemicals responsible for the toxicity [36,37]. However, to our best knowledge, there is no previous TIE study using microplastic or glitter particles.

Considering that glitter particles are globally widespread [8], and that incipient data indicate that they can be toxic to aquatic biota, ecotoxicological studies aimed at estimating their toxic potential to marine biota are required. This study evaluated the toxicity of dispersions obtained from green and white glitter particles on embryonic development of the sea urchins *Echinometra lucunter*, *Arbacia lixula*, and the mussel *Perna perna*. In addition, we used the Toxicity Identification and Evaluation (TIE phase 1) approach to identify possible chemicals related to toxicity.

## 2. Materials and Methods

### 2.1. Glitter Particles Characterization

Two types of glitter (white and green) from the same commercial brand were used in this investigation; such brand is the most sold in Brazil. These particles were previously characterized for their size distribution using laser diffraction [18]. The green glitter was composed of particles ranging between 0.002 and 0.006 mm, which could be classified as similar to medium silt, according to Wentworth’s [38] scale and Shepard’s [39] classification (Table S1). White glitter particle sizes ranged between 0.06 and 2 mm, similar to silty sand. Albanit et al. [18] also noticed that the chemical compositions of both glitters were distinct, based on information displayed on the respective glitter packages (Table S2). According to the authors, butylated hydroxytoluene (BHT), propylparaben, and talc were present in both types of glitter. In contrast, green glitter contained 12 compounds, whereas white glitter contained only 5 compounds.

The polymeric composition of white glitter was previously analyzed by Albanit et al. [18] using pyrolysis coupled with gas chromatography and mass spectrometry (Py–GC/MS), and the particles were composed of methyl acrylate–vinyl chloride (MA–VC). The authors also reported the presence of other compounds associated with PVC, such as hydrogen chloride (HCl), benzene (B), toluene (T), and anthracene (AN).

### 2.2. Glitter Dispersions’ Preparation

Glitter stock dispersions (SE500) of both glitters (green and white) were prepared by adding 500 mg of glitter particles into 1 L of filtered autoclaved seawater (dilution water) and 0.05% ethanol (to avoid particle agglomeration). The resulting mixtures (500 mg/L) were agitated during preparation of the test dispersions. Subsequently, SE500 was diluted in the dilution water to produce the following test dispersions: 50, 100, 200, 300, 400, and 500 mg/L for testing with *E. lucunter* and *A. lixula*; and 10, 25, 50, 100, 200, and 500 mg/L for the tests with *P. perna*. The experiments with *P. perna* used a different concentration range that was based on previous experiments (not shown here). A negative control consisting of 0.05% ethanol in dilution water was prepared. Thus, as reported by Albanit et al. [18], organisms should be exposed to the particles per se and the substances leached from the particles, as they are expected to occur in the natural environment. For each test dispersion, 4 replicates were prepared in glass test tubes, each containing 10 mL of test dispersion.

### 2.3. Analysis of Metals by Flame Atomic Absorption Spectrometry (FAAS)

The filtered suspension prepared with the green glitter particles was analyzed with the presence of Cu (copper), Zn (zinc), Ag (silver), Zn (zinc), Mg (magnesium), Ti (titanium), Mn (manganese), Fe (iron), and Ca (calcium). First, the SE500 was filtered through a 0.45 μm cellulose membrane to remove larger particles. Then, acidification was performed by adding nitric acid 4% to the sample and reducing the pH to 2.

The flame atomic absorption spectrometer (FAAS) equipment was calibrated. Standard stock solutions (1000 mg/L) of the studied metals were prepared using grade reagents, and serial dilutions were prepared by diluting the stock solution in deionized water. At least five concentrations were prepared for each element and three replicates were used. The minimum acceptable correlation coefficients (r^2^) in the calibration curves were 0.99 (see Supplementary Materials for details—Figures S1–S6). Calibration curves relating the measured light absorption by the spectrometer to the metal concentrations were constructed. Subsequently, the glitter samples were manually aspirated and introduced into the atomization source flame. The flame provided the necessary heat for the atomization of the metals present in the samples. During the analysis, quality control standards for Cu and Zn were included and analyzed using the same procedure used for the glitter particles, and the recoveries were 101.05% and 91.77%, respectively [40].

### 2.4. Embryonic Development Toxicity Tests with Sea-Urchin Embryos

The toxicity tests consisted of exposing the eggs of the sea urchins *Echinometra lucunter* and *Arbacia lixula* to glitter dispersions and observing their embryo development until pluteus larvae. The test method followed Brazilian standards [41], which are similar to the international protocols for sea urchin embryo tests [42,43]. *E. lucunter* and *A. lixula* are abundant in rocky reefs along the neotropical and subtropical regions, and their embryos are appropriate for testing the toxicity associated with glitter because they can be exposed to such particles (as well as other MPs) and their leachates [15]. Moreover, sea urchin embryos are sensitive to contaminants [44,45,46] and represent appropriate biological models for investigating the effects of glitter. The tests were performed according to the NBR 15,350 protocol [41]. According to the test protocol, fertilized eggs were exposed to solutions containing contaminants, and embryo development was evaluated after approximately 36–42 h for *E. lucunter* and 24–28 h for *A. lixula*.

For each experiment, approximately 20 adults of *E. lucunter* or *A. lixula* were collected from rocky reefs on the coast of São Paulo. The animals were kept in tanks containing the dilution water under constant conditions (photoperiod of 12h:12h clear–dark; temperature of 25 ± 2 °C, salinity ranging between 33 and 36, and constant gentle aeration). Spawning was induced by the injection of 0.1–0.3 mL of 1M KCl solution into the coelomic cavity of the organisms. The resulting osmotic shock stimulates the release of gametes, which are distinguished by their color: sperm is whitish, whereas ovules are reddish to purple. Gametes from at least three males and three females were used for each experiment [41]. The gametes were checked for viability under microscope, and then the fertilization was done by the addition of 0.5 mL of sperm solution to 24.5 mL of ovules solution. Fertilization success was observed by the presence of fertilization membranes. At least 90% of eggs should be fertilized for the test to be valid [41,42,43].

The tests were initiated by adding approximately 500 eggs to each test chamber (i.e., 15 mL glass tubes containing 10 mL of test dispersion). The test system was kept under controlled conditions (photoperiod of 12h:12h clear–dark; 25 ± 2 °C) during the whole test until the embryos had developed to the pluteus stage. The physicochemical parameters of the overlying solutions in the test chambers (i.e., salinity, dissolved oxygen, and pH) were measured only at the beginning of the tests because the experiments were short-term, and no significant variations were expected. The contents of each replicate were fixed by adding 10% buffered formalin (0.5 mL). The first 100 embryos of each replicate were counted under an optical microscope, and both normal larvae and those presenting any abnormalities (delayed development or morphological alterations) were counted, as recommended by the protocols [41,42,43].

### 2.5. Embryonic Development Toxicity Tests with Brown Mussel Embryos

The short-term chronic exposure assay using *P. perna* embryos followed the standard method ABNT NBR 16,456 [47] and consisted of exposing eggs of this species of mussel to the glitter dispersions and observing their development to D-larvae [47,48].

Adult mussels were collected from a mussel farm located at Cocanha Beach, Caraguatatuba, North coast of São Paulo, and taken to the laboratory. Immediately after the mussels arrived in the laboratory, spawning was induced by thermal shock [47,48]; sperm had a whitish color, and ovules were orange. The gametes were checked for integrity prior to fertilization [47,48], and the sperm solution was observed under an optical microscope to assess viability (size and movement), whereas the eggs from each female were evaluated for shape, smoothness, and size.

Similar to the test with sea-urchin embryos, the tests were conducted in glass tubes containing 10 mL of test dispersion. Four replicates were prepared for each test dispersion. In each replicate, eggs were exposed to the same test dispersions used in the experiments with sea urchin eggs and embryos. Approximately 400–500 fertilized eggs were added to each replicate, each consisting of glass test tubes containing 10 mL of the test solution. The experiment was conducted for 48 h with a photoperiod of 12 h:12 h (light:dark) and a controlled temperature (25 ± 2 °C). Embryo development was verified in experimental controls (above 70% of normal larvae, according to Zaroni et al. [48]). The test was then completed by adding 0.5 mL of 10% buffered formaldehyde to each replicate. The first 100 organisms observed in each replicate were counted and identified under a microscope. The normal veliger larvae were determined to have symmetrical and closed valves (D-shaped). Abnormal larvae included undeveloped embryos, those exhibiting delays and/or morphological abnormalities, and the absence of egg development.

### 2.6. Reference Substance Test

Reference substance tests were developed for each species to assess the sensitivity of the test batches. Sodium dodecyl sulfonate (SDS) was used for the embryonic–larval development test of *A. lixula*, *E. lucunter,* and *P. perna* at the following concentrations: 0.1, 0.5, 1, 2.5, and 5 mg/L SDS. The results were analyzed similarly to those obtained with the glitter dispersions.

### 2.7. Toxicity Identification Evaluation (TIE)

The TIE approach was originally designed to identify the toxic substances (or groups of substances) responsible for the toxicity of effluents and environmental samples of water and sediments [33,49], and was used in this study to identify the potential causes of glitter toxicity. TIE manipulations are conceptually used in a three-phase approach, in which Phase I characterizes the toxicants into main classes (characterization), Phase II identifies the specific toxicants (identification), and Phase III confirms the findings of Phases I and II (confirmation) [49,50].

Phase I involves a set of manipulations of the original sample (baseline, at 100% concentration) to modify the bioavailability and toxicity of the determined chemical groups or substances, followed by ecotoxicological assessment [33,49]. Such sample treatments involve the addition of chelating agents (such as EDTA) that form complexes with metal ions, solid-phase extraction with a C18 column to remove nonpolar organic compounds, addition of sodium thiosulfate to reduce oxidizing chemicals, filtration to remove suspended particles, aeration to remove volatile substances, pH manipulation to identify substances susceptible to pH variation, and other specific treatments directed to particular chemicals [50]. Aquatic toxicity tests were performed using the baseline and treated samples, and the results were compared. Treatments capable of altering sample toxicity indicate the respective chemical groups (or group) that influence the toxic effects. The subsequent TIE phases involve manipulation and chemical analyses to define chemicals related to toxicity [49].

The Phase I of TIE was applied, following the procedures described by the USEPA protocol [33]. To chelate divalent metals, 30 µL of EDTA (2.5% *w*/*v*) was added to each test tube, followed by a 3 h period for complete reaction and complexation. Oxidizing chemicals were removed by adding 50 µL of sodium thiosulfate (1.5% *w*/*v*) to each test tube. Sample aeration for 1 h was applied to remove volatile compounds, while filtering through a 0.45 µm membrane was used to remove solid particles. Adsorption using a C18 column was used to remove polar compounds.

### 2.8. Statistical Analyses

The data from each experiment were organized and checked for normality and variance homogeneity using the Shapiro–Wilk test and Levene’s test, respectively. The results were analyzed by analysis of variance followed by Dunnett’s test (*p* < 0.05) to compare the treatments with their respective controls, allowing for the determination of the lowest observed effect concentration (LOEC) and the no observed effect concentration (NOEC). The effective concentrations for 50% of exposed organisms were calculated using the Probits method, except for the case of green glitter and *P. perna* embryos, for which the trimmed Spearman–Karber method was employed [51]. The Paleontological Statistical 4.03—PAST and STATISTICA 6.0 packages [52,53] were used for the data analysis.

## 3. Results

### 3.1. Chemical Analysis

The green glitter was evaluated for the presence of metals (Ag, Ca, Fe, Mg, Mn, Ti, and Zn), as shown in Table 1. The concentrations of Ag in the filtered dispersion exceeded the Brazilian legal standards for marine waters [54], indicating that green glitter particles can release metals in the overlying waters.

### 3.2. Glitter Toxicity

The sensitivity of the test-organisms was assessed by the test with a reference substance (SDS), and the results indicated sensitivities within the range for the species (see Supplementary Material, Tables S3–S5).

**Figure 1:**
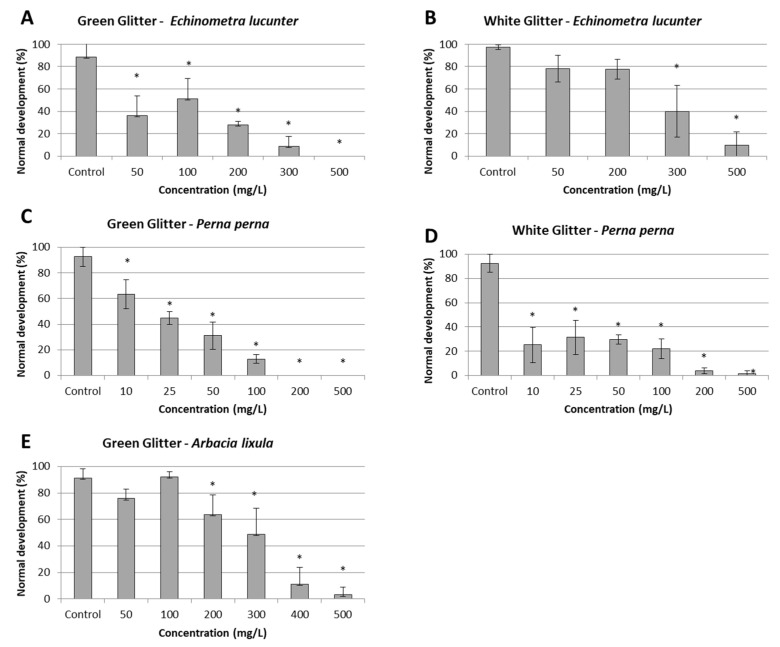
Normal embryonic development of *Echinometra lucunter*, *Perna perna,* and *Arbacia lixula* exposed to dispersions of green and white glitters. Asterisks indicate significant differences in relation to the control (*p* < 0.05). (**A**,**B**): *E. lucunter* embryos exposed to green and white glitter, respectively; (**C**,**D**): *P. perna* embryos exposed to green and white glitter, respectively; and (**E**): *A. lixula* embryos exposed to green glitter. Error bars indicate standard deviations.

The results of the toxicity tests with both types of glitter are shown in Figure 1 and the Supplementary Material (Tables S6–S11), and are summarized in Table 2. The physicochemical parameters of the tested glitter suspensions were within acceptable ranges, as shown in the Supplementary Material (Tables S12–S17). In general, dose–response curves were observed; however, for green glitter, the curves were slightly bimodal, with lower effects observed at 100 mg/L for *A. lixula* and *E. lucunter* embryos (Figure 1). However, the effects for sea-urchin embryos occurred only at high concentrations, which are unlikely to occur in the environment, at least in the near future. At low concentrations, some effects were observed on *P. perna* embryos (Figure 1; Table 2).

The dispersions prepared from both glitter colors were toxic to all tested organisms, but toxicity levels varied according to the species. The embryos of *P. perna* were more sensitive to both types of glitter, and the NOECs were lower than 10 mg/L. For this species, EC50 was not calculated for white glitter suspensions, as all concentrations affected more than 50% of the embryos. The white glitter suspension was less toxic to *E. lucunter* embryos than the green glitter suspension (Figure 1, Table 2). Green glitter was also tested with *A. lixula* embryos, which were less sensitive than *E. lucunter* and *P. perna* embryos (Table 2).

**Figure 2:**
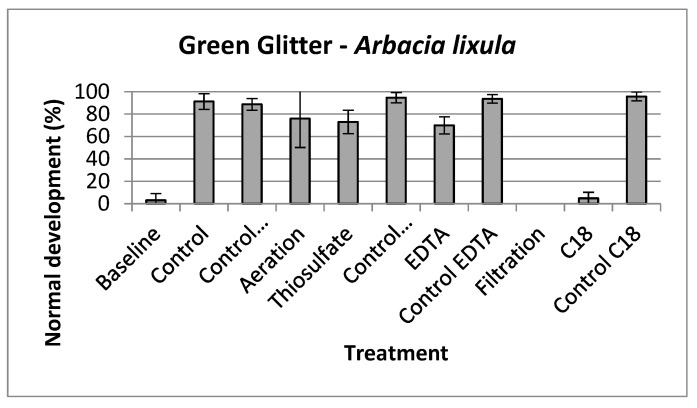
Normal development of embryos of the sea-urchin *Arbacia lixula* during the tests of the Toxicity Identification and Evaluation approach. In this experiment, samples were tested only at 100% concentrations.

The toxicity identification and evaluation approach showed that the treatments involving aeration, thiosulfate, and EDTA were capable of reducing the toxicity of the suspensions prepared with green glitter (Figure 2), suggesting that volatiles, oxidant substances, and metals contributed to the toxic effects observed. The other TIE treatments did not affect toxicity.

## 4. Discussion

Glitter particles consist of particular types of microplastics in addition to their metal layers [23], and they have been reported as environmental contaminants. Recently, oyster and mussel individuals collected from mollusk farms located in southern Brazil presented glitter particles in their soft tissues [55]. According to Campanale et al. [56], microplastics can contain two types of chemicals: (i) additives and polymeric raw materials originating from plastics, and (ii) chemicals absorbed from the surrounding environment. In the case of the glitter used in the present study, only chemicals from the first group were relevant. In this sense, our results show that glitter may release metals into the overlying water, reinforcing that glitter may be a geochemical carrier of potential contaminants. Capolupo et al. [57] showed that leachates from microplastics presented a range of substances that were capable to cause adverse effects on the bivalve *Mytilus galloprovincialis*.

Moreover, the composition of both white and green glitters, given by both Py–GC/MS analysis and manufacturer information, provides information about substances that can be potentially leached (Table S2), although such a list of potential substances can be larger, as addressed by Albanit et al. [18]. The release of chemical substances from plastics has been reported previously [58,59,60,61,62,63]. The chemicals listed in the glitters studied herein included a set of compounds such as propylparaben (PPB), butylated hydroxytoluene (BHT), methyl paraben (MeP), methyl acrylate (MA), benzene, and toluene, in addition to the Ag measured in the filtrates.

PPB has been used to extend the shelf life of personal care products [18], but can leach into the water and cause endocrine disruption in exposed organisms [64], leading to a reduction in reproductive rates and embryo development. PPB affects the growth, reproduction, physiology, and gene expression of the nematode, *Caenorhabditis elegans* [65]. In turn, BHT has been used as an additive to improve the durability of plastics. Although it is considered safe for humans at the levels used, there is some controversy regarding its toxicity to aquatic organisms [66], as it has been reported to be toxic to zebrafish embryos (*Danio rerio*) at low concentrations [66,67]. Albanit et al. [18] stated that BHT likely cause similar effects on marine organisms. MeP is widely used in cosmetic products [68] and is considered toxic to both zebrafish larvae and adults [69], as it can cause oxidative stress and endocrine disruption [70]. Methyl acrylate (MA), identified by Py–GC/MS in white glitter [18], has also been reported to be toxic to fish and invertebrates [71]. These authors observed lethal effects on rainbow trout (*Oncorhynchus mykiss*)*,* sheepshead minnow (*Cyprinodon variegatus*), *Daphnia magna*, and *Mysidopsis bahia*. Two other compounds identified in the white glitter, benzene and toluene, were described as toxic for the fathead minnow *Pimephales promelas* [72], especially during the first stages of development. Ag ions and Ag-based compounds are known to be toxic to a range of organisms [73,74]. Specifically, the Ag ions from the AgNPs interfere with the phosphate of DNA, stimulating DNA condensation and leading to a loss of replication ability in bacterial cells [75]. Ag ions act on multiple targets within cells, leading to apoptosis [76]. Thus, the chemicals present in glitter particles could cause toxicity to exposed organisms.

In fact, our results show that the embryos of *P. perna*, *A. lixula*, and *E. lucunter* are adversely affected by exposure to both types of glitter at concentrations higher that those expected to occur in the environment nowadays. In general, *P. perna* embryos were more sensitive than sea urchin embryos (Figure 1, Table 2); the LOECs for sea urchin embryos (50–300 mg/L) are unlikely to be observed in the environment, at least in the near future. These results are comparable to those obtained by Albanit et al. [18], who analyzed the toxicity of leachates of both white and green glitters on embryos of the sand-dollar *Mellita quinquiesperforata*; these authors also observed a high variability of toxicity, which was attributed to some factors, such as the particles’ aggregation, which influences the amount of leached substances in the water. Previous studies have reported that microplastics often form aggregates in aquatic environments, although such phenomena are not well understood [77,78]. Microplastic aggregation likely determines their behavior and overall fate in aquatic environments [79], including the release of substances and associated toxicity. Because microplastics aggregation may be influenced by multiple factors [77], the resulting toxicity to aquatic biota can vary widely [80,81]. Moreover, differences in particle sizes may have influenced the dispersion preparation and toxicity, as reported by Albanit et al. [18]; white glitter was easily mixed in the seawater, whereas green glitter formed a layer on the water surface.

Our results corroborate recent studies on the toxicity of glitter. Piccardo et al. [22] analyzed the toxicity associated with leachates of different types of glitter, showing worse effects on marine organisms, such as embryos of the sea-urchin *Paracentrotus lividus*. Exposure to glitter also caused oxidative stress in adults of the mussel *Mytillus galloprovincialis* [24] and brine shrimp *Artemia salina* [2]. Green et al. {32] compared the toxicity of leachates obtained from different types of glitter and observed toxicity to *Lemma minor*, regardless of the type of glitter, and the effects were attributed to the substances present in the leachates.

In our study, both glitter dispersions caused toxic effects on the embryos of the species tested, possibly because the chemicals leached in the aqueous medium. The TIE approach showed that volatiles, oxidant substances, and metals contributed to the observed toxic effects. This result is supported by chemical information, as benzene and toluene are volatile and toxic [72], and the toxicity of Ag is widely known, as previously discussed in this manuscript [74]. In addition, the degradation of plastics in aqueous media can generate oxidant substances and reactive oxygen species [82,83], as well as the degradation of some associated organic compounds, such as parabens [84]. Our results suggested that the chemical reactions occurring in glitter particles (and likely other plastic particles) immersed in water may generate toxic by-products, and this process needs to be further investigated and understood.

## 5. Conclusions

At very high concentrations, glitter dispersions were capable of causing adverse effects on the embryonic development of the mussel *P. perna* and the sea urchins *E. lucunter* and *A. lixula*; therefore, glitter particles may potentially affect other marine organisms. Glitter toxicity is primarily explained by metals (such as Ag), volatiles, and oxidant compounds, which may be present in plastics or formed during the plastic degradation process. Further studies are required to assess the potential ecological risks associated with glitter particles, especially at realistic environmental concentrations.

## Figures and Tables

**Table 1:** Concentration of metals in the filtered dispersion obtained from the green glitter SE500 and the comparison with Brazilian legal standards (CONAMA—see [54]) for marine waters and sediments. Bold values indicate exceedance of legal standards for marine waters.

Element	Concentration (mg/L)		
	Glitter	Marine Water	Effluents
Ag	**0.01**	0.005	0.1
Zn	0.014	0.09	5.0
Mg	0.25	-	-
Ti	0.001	-	-
Mn	0.008	0.1	1.0
Fe	0.018	0.3	15
Ca	0.27	-	-

**Table 2:** Effect concentration to 50% organisms (EC50), no observed effect concentration (NOEC), and lowest observed effect concentration (LOEC) of white and green glitter particles to embryos of *Arbacia lixula*, *Perna perna,* and *Echinometra lucunter*. All concentrations expressed in mg/L.

Glitter Color	*Arbacia lixula*			*Perna perna*			*Echinometra lucunter*		
	EC50	NOEC	LOEC	EC50	NOEC	LOEC	EC50	NOEC	LOEC
White	not analyzed			NC	<10	10	272.2 (261.5–282.9)	200	300
Green	246.1 (235.8–256.4)	100	200	23 (20.2–25.8)	<10	10	105.9 (61.2–150.2)	<50	50

## Data Availability

The raw data are available in the Supplementary Material.

## Supplementary Materials

The following supporting information can be downloaded at: https://www.mdpi.com/article/10.3390/toxics11070617/s1, Table S1. Grain-size distribution and classification of green and white glitter particles; Table S2. Composition of the glitters transcribed from the labels of each package. (CI 77019 = mica; CI 77891 = Titanium Dioxide); Table S3—Result of the toxicity test with reference substance (Sodium Dodecyl Sulfonate—SDS) and embryos of *Arbacia lixula*. The calculated EC50 was 1.56 (1.00–2.12) mg/L; Table S4—Result of the toxicity test with reference substance (Sodium Dodecyl Sulfonate—SDS) and embryos of *Echinometra lucunter*. The calculated EC50 was 2.31 (1.97–2.63) mg/L; Table S5—Result of the toxicity test with reference substance (Sodium Dodecyl Sulfonate—SDS) and embryos of *Perna perna*. The calculated EC50 was 1.27 (0.70–1.84) mg/L; Table S6—Result of the toxicity test with Green glitter and embryos of *Arbacia lixula*; Table S7—Result of the toxicity test with Green glitter and embryos of *Echinometra lucunter*; Table S8—Result of the toxicity test with Green glitter and embryos of *Perna perna*; Table S9—Result of the toxicity test with White glitter and embryos of *Echinometra lucunter*; Table S10—Result of the toxicity test with White glitter and embryos of *Perna perna*; Table S11—Result of the TIE approach using the Green glitter and embryos of *Arbacia lixula*. Gray cells indicate outliers; Table S12—Physical-chemical parameters of the test-suspensions during the toxicity test of green glitter using embryos of *Arbacia lixula*; Table S13—Physical-chemical parameters of the test-suspensions during the toxicity test of green glitter using embryos of *Perna perna*; Table S14—Physical-chemical parameters of the test-suspensions during the toxicity test of green glitter using embryos of *Echinometra lucunter*; Table S15—Physical-chemical parameters of the test-suspensions during the toxicity test of white glitter using embryos of *Perna perna*; Table S16—Physical-chemical parameters of the test-suspensions during the toxicity test of white glitter using embryos of *Echinometra lucunter*; Table S17—Physical-chemical parameters of the test-suspensions during the toxicity test of green glitter using embryos of *Arbacia lixula*, in the TIE approach; Figure S1—Calibration curve obtained for Ag during the chemical analysis; Figure S2—Calibration curve obtained for Ca during the chemical analysis; Figure S3—Calibration curve obtained for Fe during the chemical analysis; Figure S4—Calibration curve obtained for Mg during the chemical analysis; Figure S5—Calibration curve obtained for Zn during the chemical analysis; Figure S6—Calibration curve obtained for Cu during the chemical analysis.
