# Supplementary material for: A Glow before Darkness: Toxicity of Glitter Particles to Marine Invertebrates
Source: Toxics. 2023 Jul 16;11(7):617. doi: 10.3390/toxics11070617 (PMC10385617; doi:10.3390/toxics11070617)
Supplement: Supplementary file 1 [file toxics-11-00617-s001.zip › toxics-2497189-supplementary.pdf]

A glow before darkness: toxic effects of glitter particles to marine invertebrates

Denis Moledo de Souza Abessa[1*], Caio Cesar-Ribeiro[1,3]; Letícia França Albanit[1]; Pedro Henrique Paixão de Moura[1]; Vitória Soares Nogueira[1], Felipe Teixeira Santana[1,2], Kainã Fagundes[2], Maysa Ueda de Carvalho[1], Otto Patrão de Oliveira Muller[3]

Supplementary Material

Table S1. Grain-size distribution and classification of green and white glitter particles.

Grain size distribution statistics (Phi)	Glitter type	
	**Green**	**White**
Mean diameter	5.52	3.94
Standard deviation	0.94	0.84
Skewness	0.59	0.90
Kurtosis	3.53	4.19
**Particle size range (Wenthworth)**		
Medium sand (250 - 600 µm)	0.00	0.32
Fine sand(125 - 250 µm)	0.00	17.28
Very fine sand (63 - 125 µm)	4.24	43.93
Coarse silt (31 - 63 µm)	29.51	26.62
Medium silt (15.6 - 31 µm)	39.21	7.95
Fine silt (7.8 - 15.6 µm)	20.66	3.12
Very fine silt (3.9 - 7.8 µm)	4.94	0.73
Clay (<0.06 µm)	1.43	0.11

Table S2. Composition of the glitters transcribed from the labels of each package. (CI 77019 = mica; CI 77891 = Titanium Dioxide).

Glitter type	Green Glitter	White Glitter
**Formulation components**	BHT<br>Propylparaben<br>Talc<br>Paraffinum Liquidum<br>Methylparaben<br>Caprylic/Capric Triglycerde<br>Hydrolyzed collagen<br>Magnesium Carbonate<br>Cyclomethicone<br>Serica Powder<br>Zinc Stearate<br>Dimethicone Crosspolymer	BHT<br>Propylparaben<br>Talc<br>CI 77019<br>CI 77891

Table S3 – Result of the toxicity test with reference substance (Sodium Dodecyl sulfonate – SDS) and embryos of *Arbacia lixula*. The calculated EC50 was 1.56 (1.00 – 2.12) mg/L.

Concentration (mg/L)	Normal development (%)					
	R1	R2	R3	R4	Mean	SD
Control	93	96	81	95	91.3	6.9
0.5	82	82	68	64	74.0	9.4
1	62	80	82	75	74.8	9.0
2	0	4	3	28	8.8	12.9
3	0	0	0	0	0	0
5	0	0	0	0	0	0

Table S4. – Result of the toxicity test with reference substance (Sodium Dodecyl sulfonate – SDS) and embryos of *Echinometra lucunter*. The calculated EC50 was 2.31 (1.97 – 2.63) mg/L.

Concentration (mg/L)	Normal development (%)					
	R1	R2	R3	R4	Mean	SD
Control	98	98	92	95	95.8	2.9
0.5	99	98	94	92	95.8	3.3
1	99	100	98	98	98.8	1.0
2	40	58	70	78	61.5	16.5
3	2	4	23	4	8.3	9.9
5	0	0	0	0	0	0

Table S5 – Result of the toxicity test with reference substance (Sodium Dodecyl Sulfonate – SDS) and embryos of *Perna perna*. The calculated EC50 was 1.27 (0.70 – 1.84) mg/L.

Concentration (mg/L)	Normal development (%)					
	R1	R2	R3	R4	Mean	SD
Control	98	95	99	96	92.5	7.3
0.5	40	73	72	56	69.8	10.2
1	19	41	12	42	63.0	7.3
2	40	33	29	12	44.0	8.5
3	0	0	0	0	20.0	15.3
5	0	0	0	0	0	0

Table S6 – Result of the toxicity test with Green glitter and embryos of *Arbacia lixula*.

Concentration (mg/L)	Normal development (%)					
	R1	R2	R3	R4	Mean	Standard Deviation
Control	93	96	81	95	91.3	6.9
50	78	66	83	76	75.8	7.1
100	97	88	93	91	92.3	3.8
200	43	64	76	72	63.8	14.7
300	40	28	74	53	48.8	19.7
400	29	6	9	0	11.0	12.6
500	12	0	0	0	3.0	6.0

Table S7 – Result of the toxicity test with Green glitter and embryos of *Echinometra lucunter*.

Concentration (mg/L)	Normal development (%)				
	R1	R2	R3	Mean	Standard Deviation
Control	73	98	95	88.7	13.7
50	16	45	48	36.3	17.7
100	41	72	41	51.3	17.9
200	24	31	28	27.7	3.5
300	1	18	7	8.7	8.6
500	0	0	0	0	0

Table S8 – Result of the toxicity test with Green glitter and embryos of *Perna perna*.

Concentration (mg/L)	Normal development (%)					
	R1	R2	R3	R4	Mean	Standard Deviation
Control	97	98	93	82	92.5	7.3
10	70	73	62	48	63.3	11.2
25	45	49	38	47	44.8	4.8
50	21	29	28	46	31.0	10.6
100	9	17	12	13	12.8	3.3
200	0	0	0	0	0	0
500	0	0	0	0	0	0

Table S9 – Result of the toxicity test with White glitter and embryos of *Echinometra lucunter*.

Concentration (mg/L)	Normal development (%)				
	R1	R2	R3	Mean	Standard Deviation
Control	98	95	99	97.3	2.1
50	73	70	92	78.3	11.9
200	87	76	70	77.7	8.6
300	63	17	40	40.0	23.0
500	7	0	23	10.0	11.8

Table S10 – Result of the toxicity test with White glitter and embryos of *Perna perna*.

Concentration (mg/L)	Normal development (%)					
	R1	R2	R3	R4	Mean	Standard Deviation
Control	97	98	93	82	92.5	7.3
10	44	11	17	29	25.3	14.6
25	35	16	26	49	31.5	14.0
50	35	26	28	30	29.8	3.9
100	32	12	23	21	22.0	8.2
200	1	7	4	4	4.0	2.4
500	1	5	0	0	1.5	2.4

Table S11 – Result of the TIE approach using the Green glitter and embryos of *Arbacia lixula*. Gray cells indicate outliers.

**Treatment**	Normal development (%)					
	R1	**R2**	R3	R4	Mean	Standard Deviation
**Baseline**	12	0	0	0	3.0	6.0
**Control**	93	96	81	95	91.3	6.9
**Control alcohol**	90	91	81	93	88.8	5.3
**Aeration**	17	88	50	90	76.0	25.7
**Thiosulfate**	85	67	67	21	73.0	10.4
**Control Thiosulfate**	99	98	89	93	94.8	4.6
**EDTA**	76	77	65	62	70.0	7.6
**Control EDTA**	95	96	96	88	93.8	3.9
**Filtration**	0	0	0	0	0.0	0.0
**C18**	0	0	9	10	4.8	5.5
**Control C18**	99	91	94	99	95.8	3.9

Table S12. Physical-chemical parameters of the test-suspensions during the toxicity test of green glitter using embryos of *Arbacia lixula*.

Treatment	pH		Sal		OD (mg/L)	
	Inicial	Final	Inicial	Final	Inicial	Final
**Control water**	7.92	7.61	35	36	4.58	4.33
**Control alcohol**	7.80	7.77	35	35	4.12	4.02
**50**	7.77	7.61	35	36	4.58	4.11
**100**	7.64	7.38	35	35	4.43	4.09
**200**	7.61	7.63	34	36	4.76	4.55
**300**	7.60	7.66	34	36	4.35	4.13
**400**	7.60	7.71	34	35	5.92	5.21
**500**	7.57	7.59	35	36	4.11	4.01

Table S13. Physical-chemical parameters of the test-suspensions during the toxicity test of green glitter using embryos of *Perna perna*.

Treatment	pH		Sal		OD (mg/L)	
	Inicial	Final	Inicial	Final	Inicial	Final
**Control**	7.46	7.36	35	35	6.26	5.12
**10**	7.20	7.20	35	35	6.16	5.18
**25**	7.07	7.00	35	35	6.11	4.93
**50**	7.37	7.29	35	35	6.04	6.02
**100**	7.54	7.60	35	35	5.88	6.10
**200**	7.48	7.55	35	35	5.93	5.99
**500**	7.28	7.36	35	35	6.35	5.12

Table S14. Physical-chemical parameters of the test-suspensions during the toxicity test of green glitter using embryos of *Echinometra lucunter*.

Treatment	pH		Sal		OD (mg/L)	
	Inicial	Final	Inicial	Final	Inicial	Final
**Control**	7.98	8.01	34	34	6.46	4.32
**50**	7.38	7.91	34	34	5.88	4.11
**100**	7.63	7.66	34	34	5.92	4.11
**200**	7.37	7.42	34	34	5.93	4.12
**300**	7.54	7.33	34	34	6.04	4.12
**500**	7.48	7.25	34	34	6.10	4.13

Table S15. Physical-chemical parameters of the test-suspensions during the toxicity test of white glitter using embryos of *Perna perna*.

Treatment	**pH**		**Sal**		**OD (mg/L)**	
	**Inicial**	**Final**	**Inicial**	**Final**	**Inicial**	**Final**
**Control**	7.46	7.36	35	35	6.26	5.12
**10**	7.35	7.02	35	35	6.20	5.13
**25**	7.41	7.99	35	35	6.04	5.19
**50**	7.52	7.88	35	35	5.93	5.22
**100**	7.66	7.90	35	35	6.10	4.98
**200**	7.42	7.12	35	35	5.99	4.66
**500**	7.82	7.55	35	35	6.02	5.99

Table S16. Physical-chemical parameters of the test-suspensions during the toxicity test of white glitter using embryos of *Echinometra lucunter*.

Treatment	**pH**		**Sal**		**OD (mg/L)**	
	**Inicial**	**Final**	**Inicial**	**Final**	**Inicial**	**Final**
**Control**	7.98	8.01	34	34	6.46	4.32
**50**	7.85	7.98	34	35	6.12	4.33
**200**	7.41	7.33	34	34	6.26	4.35
**300**	7.68	7.66	34	34	5.44	4.43
**500**	7.58	7.55	34	34	5.31	4.51

Table S17. Physical-chemical parameters of the test-suspensions during the toxicity test of green glitter using embryos of *Arbacia lixula*, in the TIE approach

Treatment	**pH**		**Sal**		**OD (mg/L)**	
	**Inicial**	**Final**	**Inicial**	**Final**	**Inicial**	**Final**
**Control**	7.92	7.61	35	36	4.58	4.33
**Control alcohol**	7.80	7.77	35	35	4.12	4.02
**Control Thiosulfate**	7.97	7.85	35	35	5.14	5.12
**Thiosulfate**	7.66	7.60	35	35	5.22	4.51
**Control EDTA**	7.90	7.66	35	35	4.20	4.23
**EDTA**	7.47	7.60	35	35	5.14	4.66
**Control C18**	7.57	7.32	35	35	4.88	4.32
**C18**	7.71	7.57	35	35	4.99	5.01
**Filtration**	7.42	8.08	35	35	4.66	4.12
**Aeration**	7.60	7.58	35	35	5.96	4.98

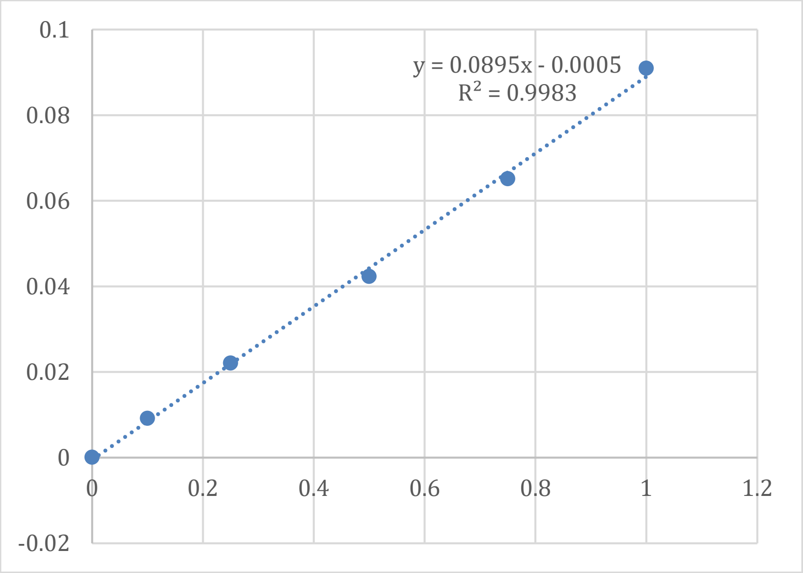

Figure S1. Calibration curve obtained for Ag during the chemical analysis.

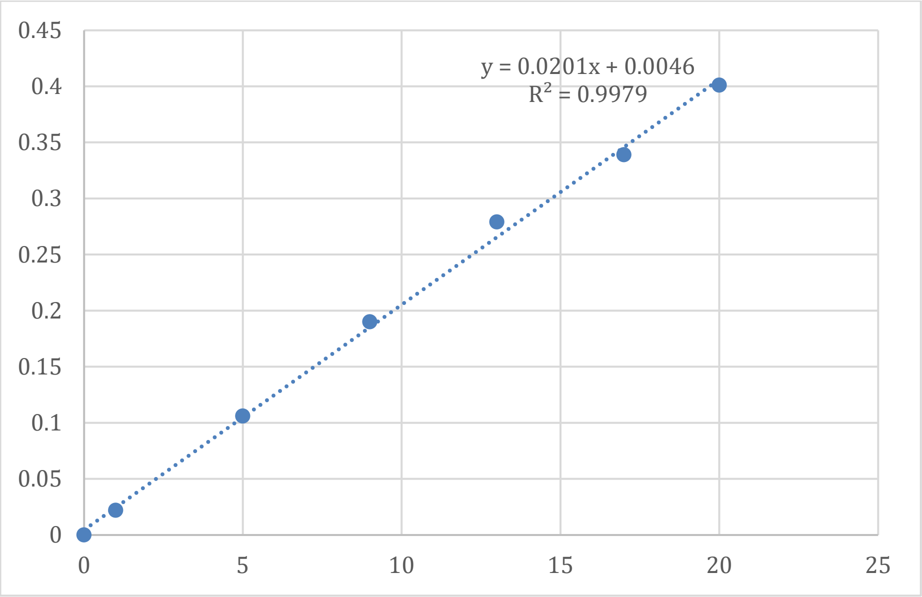

Figure S2. Calibration curve obtained for Ca during the chemical analysis.

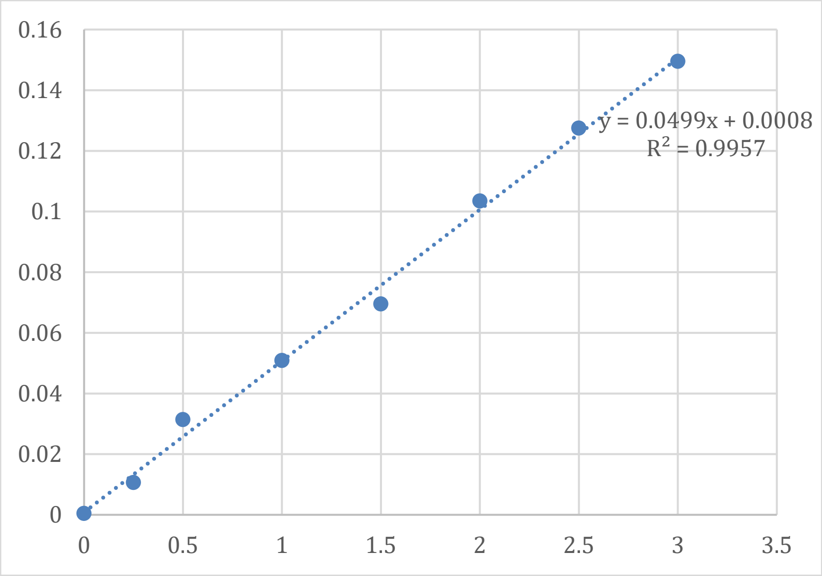

Figure S3. Calibration curve obtained for Fe during the chemical analysis.

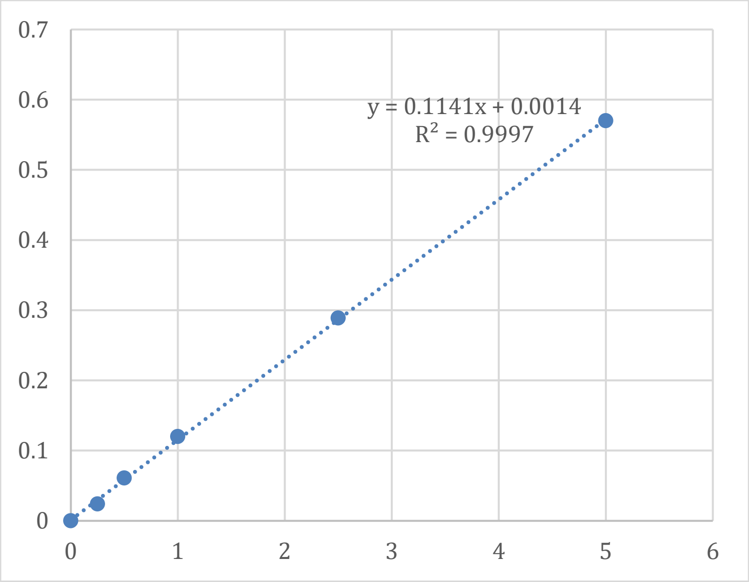

Figure S4. Calibration curve obtained for Mg during the chemical analysis.

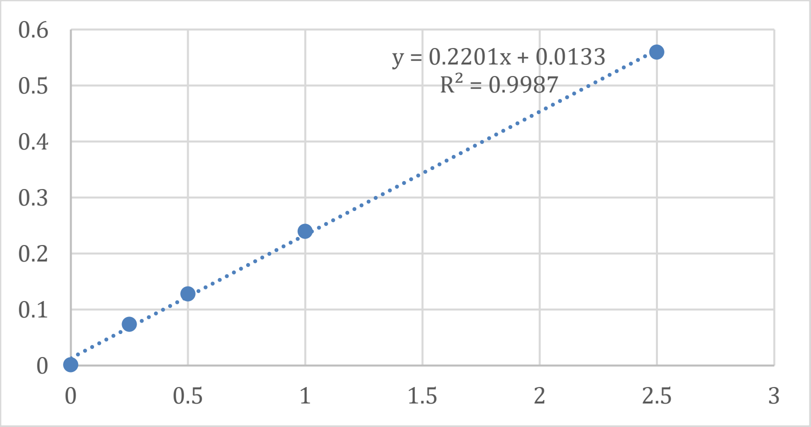

Figure S5. Calibration curve obtained for Zn during the chemical analysis.

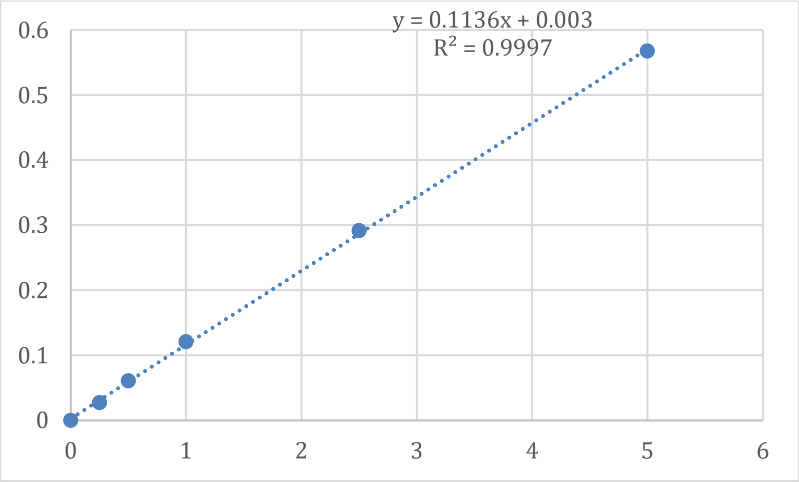

Figure S6. Calibration curve obtained for Cu during the chemical analysis.
